# Evaluations of high-viscosity glass-ionomer cement modified by eucalyptus extract-loaded mesoporous silica nanoparticles: in vitro study

**DOI:** 10.1007/s10266-025-01291-2

**Published:** 2026-01-20

**Authors:** Mai Samy Sheta, Zienab E. Eldin

**Affiliations:** 1 Dental Biomaterials Department, Faculty of Dentistry, Tanta University, El-Giesh St., Tanta, Gharbia Egypt; 2 Materials Science and Nanotechnology Department, Faculty of Postgraduate Studies for Advanced Sciences, Beni-Suef University, Beni-Suef, 62511 Egypt

**Keywords:** High-viscosity glass-ionomer cement, Antimicrobial properties, Eucalyptus extract, Mechanical properties, Fluoride release

## Abstract

Glass-ionomer cements offer unique qualities, including bulk insertion, chemical adhesion to tooth tissues, fluoride release, as well as recharge**.** This study aimed to assess the effect of addition of eucalyptus extract-loaded mesoporous silica nanoparticles (EE@MSNs) to high-viscosity glass-ionomer cement HVGIC on its mechanical properties, antimicrobial activity, and fluoride release. EE@MSNs were prepared and characterized. Then, they were added to HVGIC Fuji IX at weight percentages of 1%, 3%, and 5% to create three experimental groups which were compared with the unmodified control group. Compressive strength, Vickers microhardness, and antimicrobial activity against *Streptococcus mutans* were evaluated. Fluoride release was assessed after 24 h and 7 days. HVGIC modified by 1% and 3% EE@MSNs recorded significant improvement in compressive strength and Vickers microhardness compared to control groups. While higher ratio 5% showed decline in the mechanical properties. Experimental groups recorded significant increase in antibacterial activity and fluoride release when compared to control group and this increase was proportional to EE@MSNs percentage. Addition of 1 and 3 wt.% EE@MSNs could be considered a promising approach to modify properties of HVGICs.

## Introduction

Glass-ionomer cement (GIC) is a well-known restorative dental material having numerous relevant clinical uses as it has the same coefficient of thermal expansion as dentine, chemical adhesion to tooth tissues, bioactivity, and its ability to release and recharge fluoride [1–6]. Despite their numerous benefits, the GICs are rarely employed as permanent filling materials for stress-bearing regions due to their weak mechanical qualities. The material’s low fracture toughness, wear resistance, and hardness make it prone to failure under strong masticatory forces [7, 8].

For strengthening the GICs, numerous chemical alterations were investigated [9]. One technique involved regulating size of glass particles, which gave rise to a high powder-to-liquid ratio. When compared to conventional GICs, this method produced high-viscosity GICs (HVGICs) with better qualities. [8, 9].

HVGICs are perfectly suited for management of dental caries, since they may be used in the earliest phases of caries formation or even in large cavities. They simplified the restorative treatment [10]. In addition, HVGIC is the preferred choice for atraumatic restorative treatment (ART) procedure [3].

Since residual caries persists after hand excavation, the antimicrobial activity of HVGIC utilized by the ART procedure becomes more important [11]. Furthermore, the antibacterial properties of the material are critical for avoiding biofilm development and thus recurrent caries [11, 12]. Previous investigations indicated that fluoride release as well as low pH while setting contributes to the GIC’s antibacterial action [6, 13]. The initial ‘burst effect’ of release of fluoride impacts microbial enzymes necessary for cellular metabolism of the bacteria, lowers intracellular pH and produces a bacteriostatic impact, especially on *Streptococcus mutans* and *Lactobacillus acidophilus* [14]. Throughout the first months, the inherent fluoride level rapidly depletes [15]. The cement can absorb more fluoride during the service period of the restoration [15, 16]. As a result, GIC acts as a fluoride reservoir and slowly re-releases fluoride. Although the fluoride produced by GIC inhibits microorganisms in vitro, clinically it still undefined [16]. As a result, it is essential to strengthen HVGIC’s antibacterial capabilities by incorporating antimicrobial chemicals that limit the growth of caries-causing bacteria like *S. mutans*, so can enhance the therapeutic results of ART [17].

Several studies were conducted to augment the antibacterial activity by adding several antibacterial compounds to the powder or liquid of GIC. Addition of chlorhexidine (CHX) to GICs was accepted to improve their antibacterial activity [18]. However, studies have shown that its high ratios induce harm to odontoblastic cells and decrease the mechanical characteristics of GICs [19].

Yesilyurt et al. [20] evaluated GICs comprising a ciprofloxacin, metronidazole, and minocycline antibiotic combination. This modification has produced an improved antibacterial behavior but inferior physico-mechanical properties.

Some of the additives that also have been explored are triclosan, quaternary ammonium salts, and metallic dopants, such as silver, zinc, and titanium [6]. Studies have shown that they improved antimicrobial characteristics of GICs. However, all these additives have the potential to affect the oral microbiology and cause undesirable side effects like teeth discoloration, bad taste and smell, allergy, and toxicity [21].

There is widespread worry in medicine and dentistry about the growth of antibiotic-resistant microorganisms, because they constitute a substantial danger to world health, specifically oral health. The rising worry that oral infections could acquire resistance to traditional preventative methods underscores the need for alternate ways to inhibit their development without creating microbial resistance [22]. Natural products possessing antibacterial effects have gained considerable interest because of their efficacy and lack of drug resistance [23]. Studies have shown that they are promising additives that improved antimicrobial properties of GIC [24].

Eucalyptus leaf extract is an alternative to be considered as it possesses broad-spectrum antimicrobial effects against key oral pathogens [25]. It contains essential oils and phenolic compounds with high antimicrobial potential and exhibits sustained release, prolonged activity, and reduced toxicity [26, 27]. Its low cost and global availability further enhance its potential for clinical application.

Eucalyptus oil was regarded an adjuvant aid in limiting the bacteria that cause dental caries and endodontic infections [22]. Mohammed found that the methanolic extract of Eucalyptus leaves inhibited *S. mutans*, a pioneering bacteria responsible for dental caries more effectively than gentamycin and nystatin [28]. Another study confirmed that the extracts of eucalyptus twigs demonstrated strong antimicrobial effects on *S. mutans* [29]. Yet, application of eucalyptus leaf extract in dental restorative materials remains largely unexplored. It represents a novel bioactive modification of GIC. Therefore, evaluating its effect on HVGIC could offer a new pathway for developing restorative biomaterials with enhanced therapeutic function specially for ART.

HVGIC requires a modification improves the mechanical qualities and antimicrobial activity, and boosts fluoride release. This may be accomplished by the regulated release of antibacterial agent via mesoporous fillers. Mesoporous Silica Nanoparticles (MSNs), one of the various suggested encapsulation techniques, have attracted a lot of research attention because of their highly advantageous morphological characteristics, which include an extensive surface area, porous structure, biocompatibility, and attractive physicochemical qualities having the potential to function as a system for drug delivery [30].

In this study, eucalyptus extract-loaded mesoporous silica nanoparticles (EE@MSNs) were synthesized, characterized, and then added as fillers to HVGIC. The null hypothesis of this study was that (EE@MSNs) would not affect mechanical properties, antimicrobial behavior and fluoride release of HVGIC.

## Materials and methods

The details of the materials used in this investigation are listed in Table 1.

**Table 1:** Materials utilized in the study:

Category	Chemical	Manufacturer	Lot no
Eucalyptus leaves extract-loaded mesoporous silica nanoparticles (EE@MSNs)	Tetraethyl orthosilicate (TEOS)	Sigma-Aldrich	S8250158228
	(3-Aminopropyl) triethoxysilane (APTES)	Sigma-Aldrich	Pcode:102465057
	Sodium hydroxide	Loba Chemie	1310-73-2
	Hexadecyltrimethylammonium bromide (CTAB)	Loba Chemie	CAS No.: 57-09-0
	Methanol, HPLC	Merck	CAS No. 67-56-1
High-viscosity GIC (HVGIC)	Fuji IX GP glass ionomer (MINIPACK)	GC Corporation, Tokyo, Japan	Batch number: 002578

### Synthesis of eucalyptus extract-loaded mesoporous silica nanoparticles (EE@MSNs)

Eucalyptus leaves were extracted using the maceration technique with 70% methanol at room temperature for 24 h [28]. This process was repeated three times. The resulting liquid extracts were then evaporated, and the dried extracts were prepared for subsequent analysis.

Mesoporous silica nanoparticles were synthesized using reaction mixtures with reagent specific concentrations. Initially, CTAB (2.74 mM) was first dissolved in distilled water, then of NaOH (3.8 mM) was added, followed by heating the solution at 80 °C in an oil bath while stirring at 800 rpm with a magnetic stirrer. After 30 min of heating, TEOS (27.09 mM) was added drop by drop and the solution was stirred at 80 °C for another 2 h. The resultant white precipitate was extracted through vacuum filtration with a Buchner funnel and thoroughly rinsed by water. Then, it was dried in an oven at 140 °C for 2 h and crushed into a fine powder by a mortar and pestle. Finally, calcination was carried out at 550 °C for 5 h to remove the template [31–33].

Amino-Functionalized Mesoporous Silica (MSN-NH2**)** was prepared by treating MSNs with APTES. Specifically, 1 g of MSNs was blended with 200 mL of anhydrous ethanol and stirred for 5 min. Subsequently, 0.5 mL of APTES was then added, and the mixture was stirred at 80 °C for 8 h. The resulting MSNs-NH_2_ were collected through centrifugation and rinsed several times by double-distilled water. For extract loading, a weight ratio of 10:1 (MSNs to extract) was used. In detail, 0.1 g of Eucalyptus extract and 1 g of MSNs-NH_2_ were dissolved in 10 mL of ethanol while stirring at room temperature for 24 h. The mixture was then centrifuged at 10,000 rpm for 8 min. Finally, the EE@MSNs were washed repeatedly with ethanol then dried in an oven at 80 °C for 8 h [31].

### Morphological and chemical characterization of EE@MSNs

The morphology and surface topography of EE@MSNs nanocomposite were analyzed using scanning electron microscope (SEM). Imaging was conducted with a Philips-XL30 device (The Netherlands) to study the surface characteristics. The produced samples of EE@MSNs were analyzed using Fourier transform infrared spectroscopy (FTIR) by a Bruker Vertex 70 FTIR-FT Raman spectrometer. The samples were prepared by mixing them with IR-grade potassium bromide and pressing them onto disks. An absorption range of 4000–400 cm^−1^ was used to scan the spectra. The crystalline structures of the produced powder of EE@MSNs were investigated using powder X-ray diffraction (PXRD) analysis. Cu Kα radiation (*λ* = 1.54 Å) was used in the analysis to scan the powdered materials throughout a 2*θ* range of 10–70°. The device was operated at a voltage of 40 kV, a current of 30 mA, a scanning speed of 2° per minute, and a 2*θ*/*θ* scanning mode.

### Cytotoxicity evaluation of MSNs and EE@MSNs

The study evaluated samples’ toxicity using dental pulp stem cells (DPSCs). DPSCs acquired from the holding company’s tissue culture unit for biological products and vaccinations (VACSERA). The cells were plated at 1 × 10^4^ cells per well and exposed to various concentrations (7.8–1000 µg/mL) of MSNs and EE@MSNs. Cell viability was measured at 48 h using the MTS assay, with untreated DPSCs serving as controls. After adding the MTS reagent in DMEM, incubation of the plates was carried out at 37 °C for 2 h with 5% CO_2_. Cell viability percentages were calculated via measuring absorbance at 560 nm with a spectrophotometer, with all tests performed three times for reliability.

### Incorporation of EE@MSNs into glass-ionomer cement and specimen preparation

After EE@MSNs preparation, they were weighed using an electronic balance (Radwag Wagi Elektroniczne, AS 220.B, 509892, Poland) at an accuracy of 0.0001 g, and then added to glass-ionomer cement powder in percentages of (0% control and 1%, 3%, and 5% for experimental groups by the weight of the powder). The EE@MSNs were mixed with GIC powder using mechanical mixer in mechanical stirrer (5040001 RW28, Atlanta, USA) at 20 rpm speed for 1 h at room temperature. For each test, ten specimens were made per group. The specified ratio of 3.6 powder/1 liquid Fuji IX was used to prepare each specimen at room temperature. Specimens were manually mixed on a glass slab using a plastic spatula. For every test, a Teflon mold with the required measurements was made. The mold was filled with the mixture. It took 2 min and 30 s to set. By applying physical pressure to both sides of the specimen, glass microscopic slides with transparent polystyrene matrix sheets were used for extruding excess material. Specimens were removed from their mold after one hour, ground with wet silicon carbide (600-grit). Prior to mechanical properties’ evaluation, incubation of the specimens was done in distilled water for 24 h (BTC, BT1120, Biotech, Cairo, Egypt).

### Grouping

The design and procedures of the present study were accomplished according to the research guidelines adopted by the Research Ethics Committee, Faculty of Dentistry, Tanta University (**#R-BIO-9-24-3144**). The studied groups were control group which had 0% EE@MSNs and experimental groups: 1% EE@MSNs, 3% EE@MSNs, and 5% EE@MSNs. Ten specimens (*n* = 10) were made for every property for every group. The sample was gathered in accordance with earlier research [30]. A computer software called G Power version 3.1.9 was used to determine the sample size. The actual power was 96.86%, the effect size was 0.9863, the minimum sample size was 24, the significance threshold was set at 0.05, and the confidence interval was 95%. To account for any failure and boost the validity of the findings, the sample was expanded to 40. In this study, compressive strength, Vickers microhardness, antimicrobial properties, and fluoride release on 24 h and 7 days were evaluated.

### Properties’ evaluation

#### Compressive strength

For compressive strength testing, cylindrical specimens of 4 mm in diameter and 6 mm in thickness were prepared based on International Standard Organization (ISO) No. 9917-1[34] using a Teflon split mold. After keeping the specimens in distilled water 24 h, they were tested for compressive strength using Universal Testing Machine (Instron, Model: 3365, Norwood, USA) at a crosshead speed of 1.0 mm/min until the specimen fractured.

#### Vickers microhardness

A Teflon mold was used to create cylindrical specimens of 3 mm thickness and 6 mm diameter (*n* = 10). The specimens were tested after being stored in distilled water at 37 °C for 24 h. Vickers hardness measurements were conducted by a Digital Microhardness Tester (Zwick/Roell, INDENTEC, West Midlands, England). Three indentations were made for each specimen by application of 50 gf with a dwell duration of 30 s, and then, the average of them was calculated [35].

### Antimicrobial evaluation

#### Bacterial culture

*Streptococcus mutans (S. mutans)* ATCC 25175 was sourced from the American Type Culture Collection (ATCC). The bacteria were cultured on tryptic soy agar (TSA; Merck, USA) and cultivated in tryptic soy broth (TSB; Merck, USA) at 37 °C for 18 h with continuous shaking at 180 rpm.

### Determination of minimum inhibitory concentration (MIC) and minimum bactericidal concentration (MBC) of MSN and EE@MSNs on *S. mutans*

The study evaluated antimicrobial effectiveness through two key measurements. The lowest sample concentration which stopped microbial growth was identified as the MIC, while the MBC represented the minimum concentration needed to eliminate 99.9% of bacteria. A twofold dilution approach was utilized, and microorganisms were treated with various concentrations of samples in DMSO (300 mg/mL). Chlorhexidine (CHX) was used as a positive control due to its established status as a gold-standard antimicrobial agent in dental applications, with well-documented efficacy against a broad spectrum of oral pathogens, including *S. mutans*. A stock solution of 1 mg/mL was prepared for use in the assays. After 24-h anaerobic incubation at 37 °C, the highest extract concentration showing broth turbidity indicated the MIC. To determine MBC, samples showing no growth were cultured on BHI agar and incubated under identical conditions, with MBC regarded as the lowest concentration achieving 99.9% microbial elimination. The results correspond to three experiments independently.

### Agar diffusion method

The study assessed effectiveness of different tested groups against *S. mutans* through well-diffusion testing. The bacteria were first grown in nutrient broth for 24 h, and then spread across Muller-Hinton agar plates. Sterile tools were used to create the cement disks (3 mm in thickness and 6 mm in diameter), which were then sterilized by UV light (CAMAG UV Cabinet, CAMAG Germany) at 254 nm for 60 min before shifting on to the following stage [36]. The plates were incubated overnight at 37 °C to evaluate antimicrobial effect. The experiment included chlorohexidine as a positive control and DMSO as a negative control. The set specimens’ antibacterial effectiveness against *S. mutans* was evaluated by measuring inhibition zone surrounding them using a digital caliper.

### Fluoride release

Round teflon mold of 5 mm in diameter and 3 mm in thickness was used to prepare the required specimens (*n* = 10) [37]. Then, each specimen was kept in 5 mL of deionized water in a firmly sealed polyethylene test tube at 37 °C in incubator (BTC, BT1120, Biotech, Cairo, Egypt). Fluoride ion content in the deionized water was zero. After 24 h, the tubes were carefully shaken, and the specimens were withdrawn and rinsed with 1 mL of deionized water and inserted into another vial contained 5 mL of fresh deionized water which was replenished every 24 h. The above technique was repeated for 7 days. Fluoride ion concentration (µg/mL) in the deionized water was measured after 24 h and 7 days [38]. Each obtained deionized water sample was buffered by equivalent volume of TISAB II (Total Ionic Strength Adjustment Buffer solution) to stabilize pH and avoid the development of fluoride complexes consequently [39]. Fluoride ion concentrations were measured by Ion chromatograph (ICS 3000, Dionex) [40].

## Statistical analysis

Analysis of data was done by one-way ANOVA, incorporating descriptive statistics such as the means and standard deviations. Tukey’s post hoc test was utilized to compare between groups. Statistical significance of all the tests was established at *p* < 0.05. Statistical analyses were conducted by the Statistical Package for Social Sciences (SPSS version 26).

## Results

### Morphological and chemical characterization

**Fig. 1:**
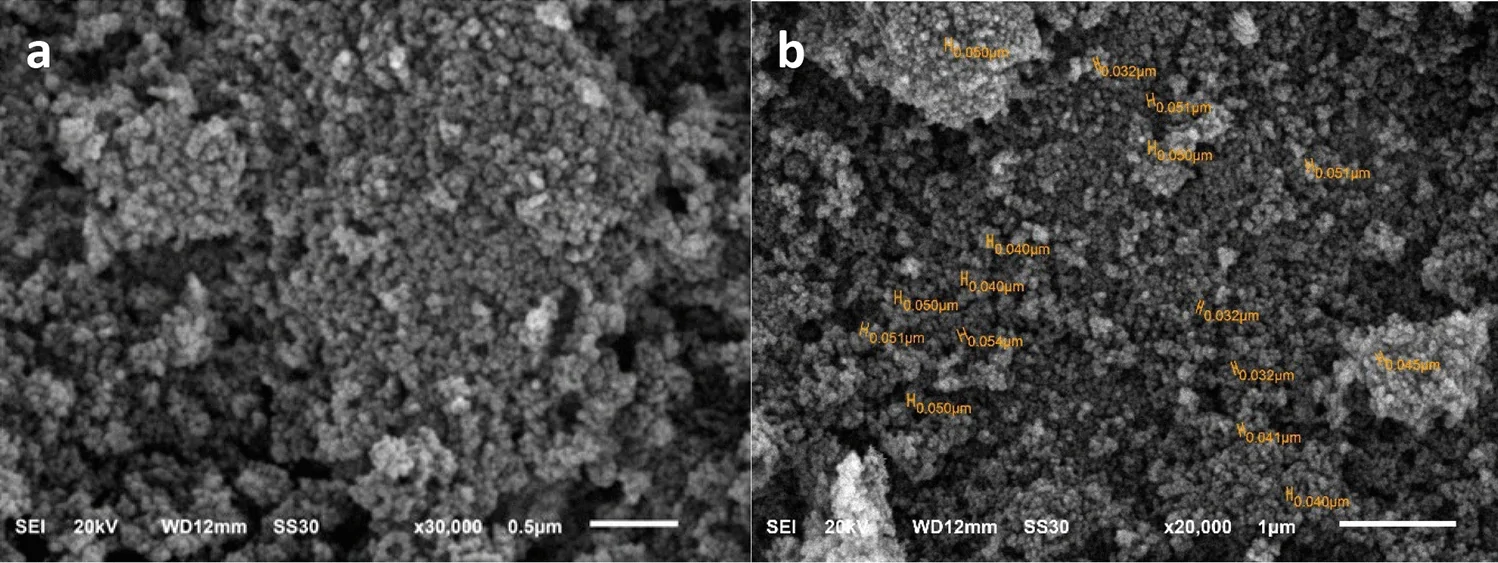
Scanning electron image of EE@MSNs: **a** at × 30,000 magnification, and **b** at × 20,000 magnification with measured sizes of nanoparticles

The SEM image of EE@MSNs (Fig. 1) revealed a highly aggregated, porous structure with particle sizes predominantly below 100 nm. The rough surface texture is indicative of successful functionalization with amine groups and the incorporation of Eucalyptus extract, suggesting effective loading of bioactive compounds. The aggregation observed may result from interparticle interactions, such as hydrogen bonding or van der Waals forces, which are common in mesoporous silica nanoparticles. This morphology aligns with MSN-NH_2_ and highlights their potential for applications in drug delivery or bioactive compound release systems.

**Fig. 2:**
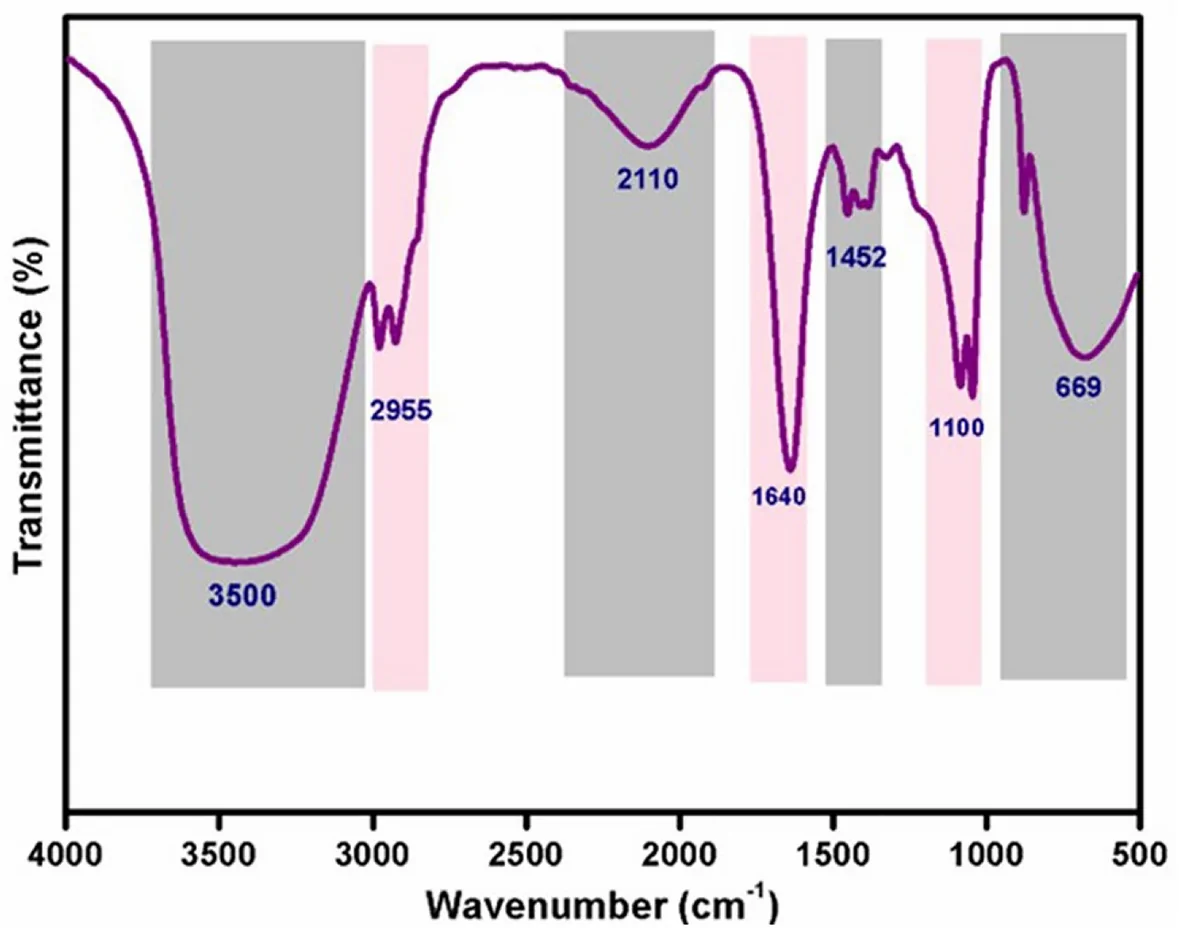
FTIR spectra of EE@MSNs

As shown in Fig. 2, the FTIR spectrum of EE@MSNs demonstrated characteristic peaks indicative of successful loading and functionalization. A broad peak at 3500 cm⁻^1^ corresponds to O–H-stretching vibrations, signifying hydroxyl groups from the extract or residual silanol groups. The peak at 2955 cm⁻^1^ represents C–H stretching, suggesting aliphatic hydrocarbons from the extract. A distinct peak at 1640 cm⁻^1^ was attributed to stretching of C=O, highlighting the presence of carbonyl groups from bioactive compounds in the extract. Peaks at 1100 cm⁻^1^ and 669 cm⁻^1^ confirm the stretching of Si–O–Si and bending vibrations, verifying the structural integrity of the silica framework. These findings collectively indicate strong interactions between the Eucalyptus extract and MSN-NH_2_, confirming successful incorporation of plant-based bioactive compounds into the functionalized nanoparticle.

**Fig. 3:**
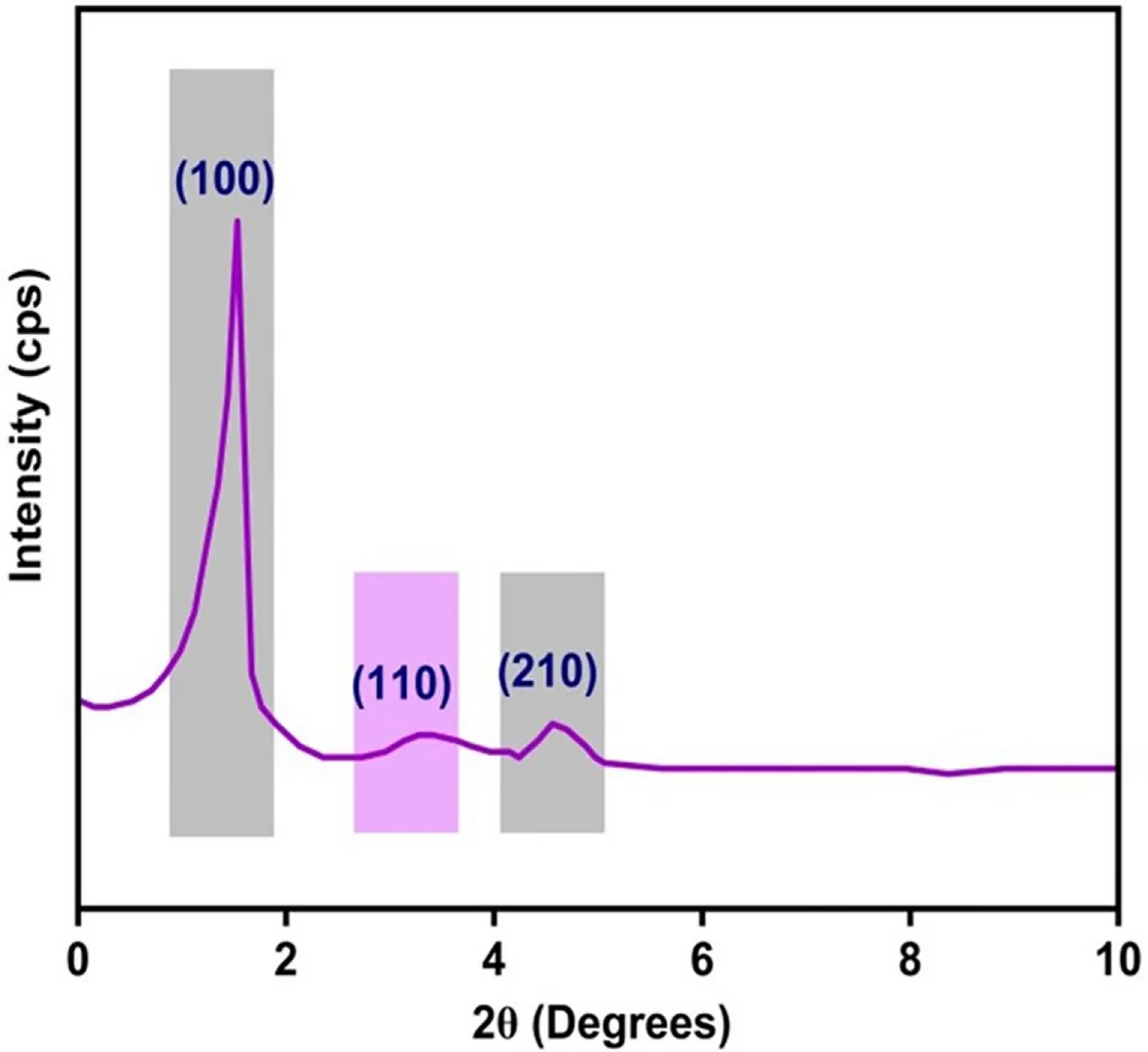
XRD of EE@MSNs

The X-ray diffraction (XRD) pattern of EE@MSNs demonstrated characteristic peaks confirming the mesoporous structure of the material (Fig. 3). The intense peak at a low angle of 2*θ* ≈ 2° corresponds to the (100) plane, a hallmark of hexagonal mesoporous silica, indicating the presence of ordered mesopores. The weaker peaks at higher angles, attributed to the (110) and (210) planes, further validate the structural integrity and periodicity of the mesoporous framework. The reduced intensity of these higher order reflections compared to the pristine MSN-NH₂ suggests partial pore filling or slight disordering due to the successful incorporation of the Eucalyptus extract into the mesopores. This incorporation likely results in a slight reduction in pore uniformity and surface area while maintaining the overall mesoporous structure, as indicated by the retention of the (100) peak. These findings confirm the effective loading of Eucalyptus extract into the MSN-NH₂ without compromising the mesoporous architecture.

### Cytotoxicity evaluation of MSNs and EE@MSNs

**Fig. 4:**
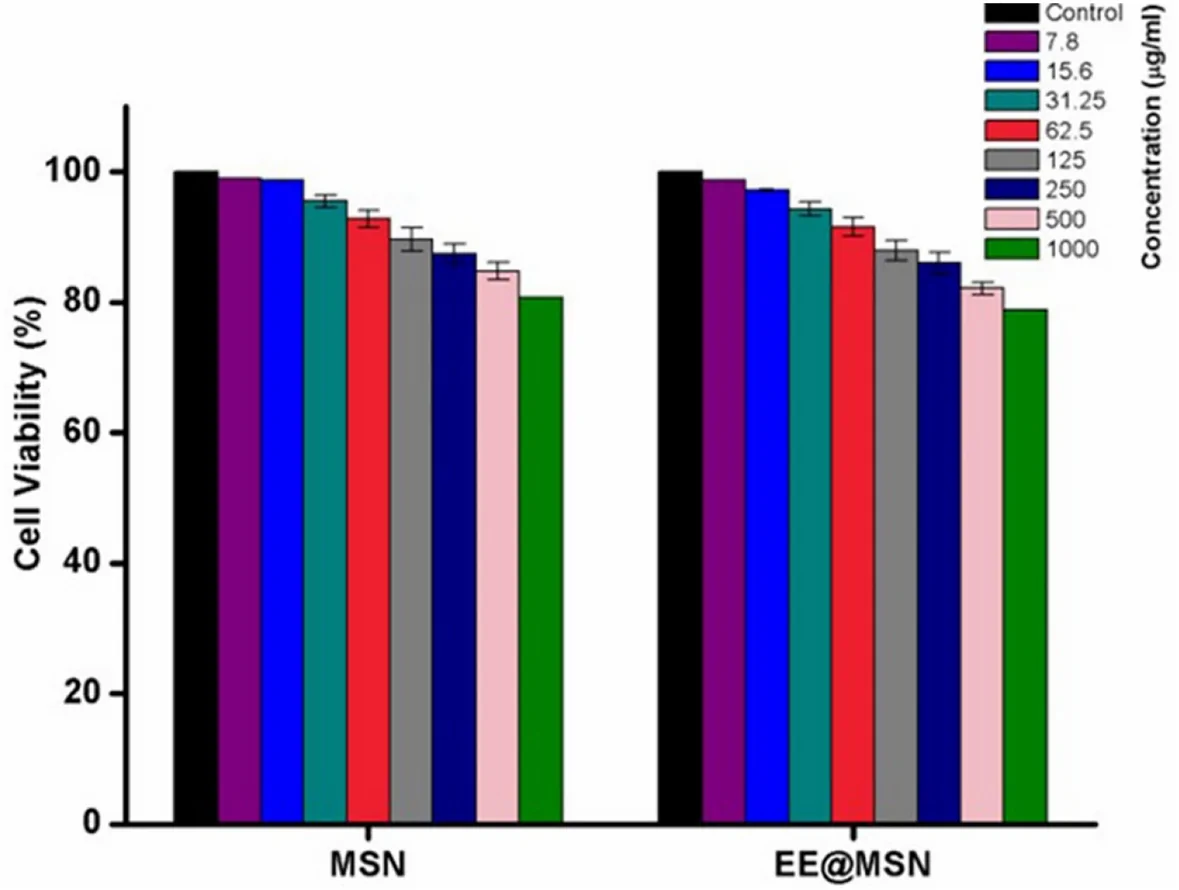
Cell viability (%) of MSN and EE@MSNs at different concentrations after 24 (*n* = 3) ± SD

As illustrated in Fig. 4, both MSNs and EE@MSNs demonstrated excellent biocompatibility. Across all tested concentrations, cell viability remained consistently above 80%, a value that is considered non-cytotoxic according to the ISO 10993-5 standard. While a minor, dose-dependent decrease in viability was noted at higher concentrations (500 and 1000 µg/mL), the ANOVA revealed that these reductions were not statistically significant (*p* > 0.05) when compared to the untreated control cells. These findings confirm that encapsulating eucalyptus extract within the MSNs does not introduce any significant cytotoxicity, affirming the safety profile of the formulation for potential biomedical applications.

### Properties’ evaluation

#### Compressive strength

**Fig. 5:**
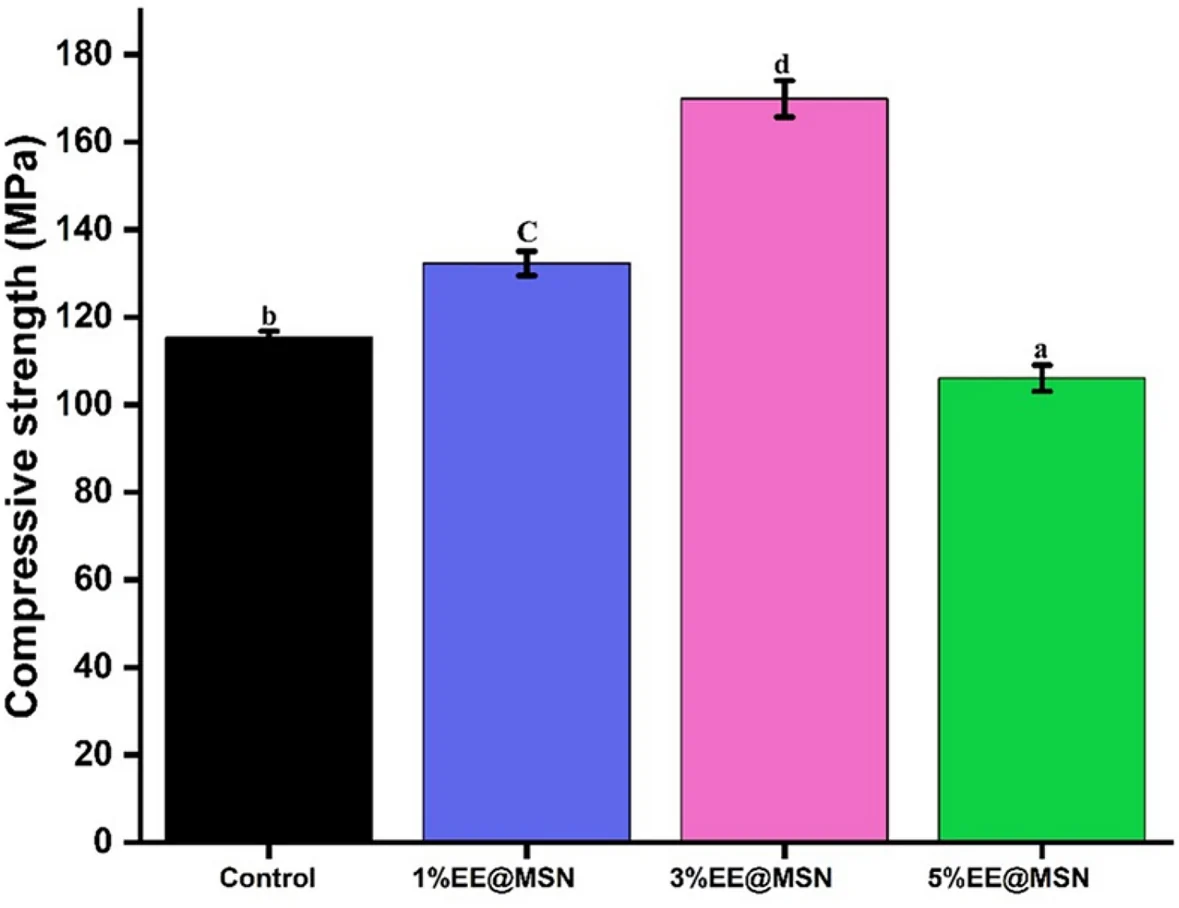
Compressive strength of different tested groups. Columns with the same letter show no significant difference at *p* < 0.05 whereas different letters showing significant difference at *p* < 0.05

Results of compressive strength testing are shown in Fig. 5. A one-way ANOVA revealed a significant improvement in compressive strength of the two experimental groups 1% EE@MSNs and 3% EE@MSNs than control group. While 3% EE@MSNs recorded significant increase in compressive strength than all other tested groups. Compressive strength of 5% EE@MSNs was significantly lower than all tested groups (*p* < 0.05).

#### Vickers microhardness

**Fig. 6:**
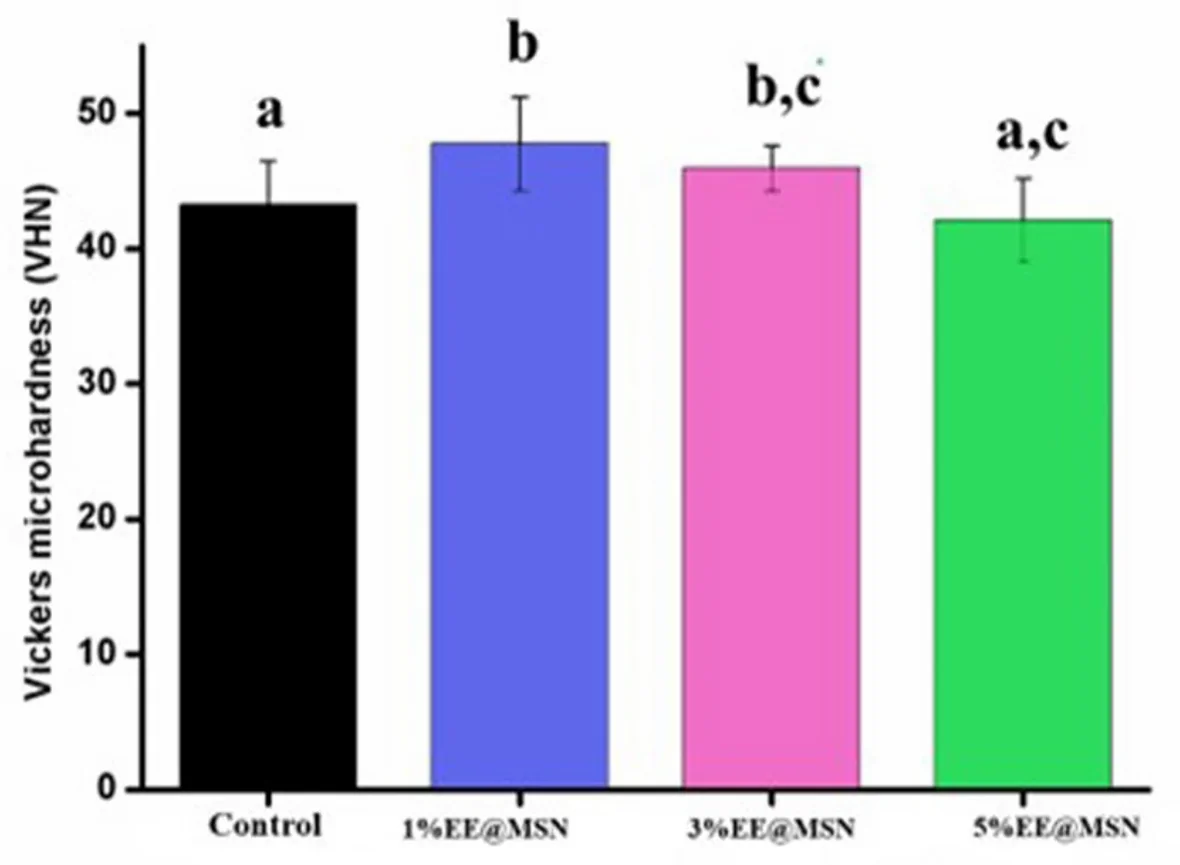
Vickers microhardness of different tested groups. Columns with the same letter show no significant difference at *p* < 0.05, whereas different letters show significant difference at *p* < 0.05

Vickers microhardness results were presented in Fig. 6. 1% EE@MSNs and 3%EE@MSNs groups recorded significantly higher microhardness than control group (*p* < 0.05), while they were not significantly different from each other. 5% EE@MSNs group showed significant decrease in microhardness when compared to 1% EE@MSNs group, while it was not significantly different than other tested groups.

### Antimicrobial evaluation

#### MIC & MBC

**Fig. 7:**
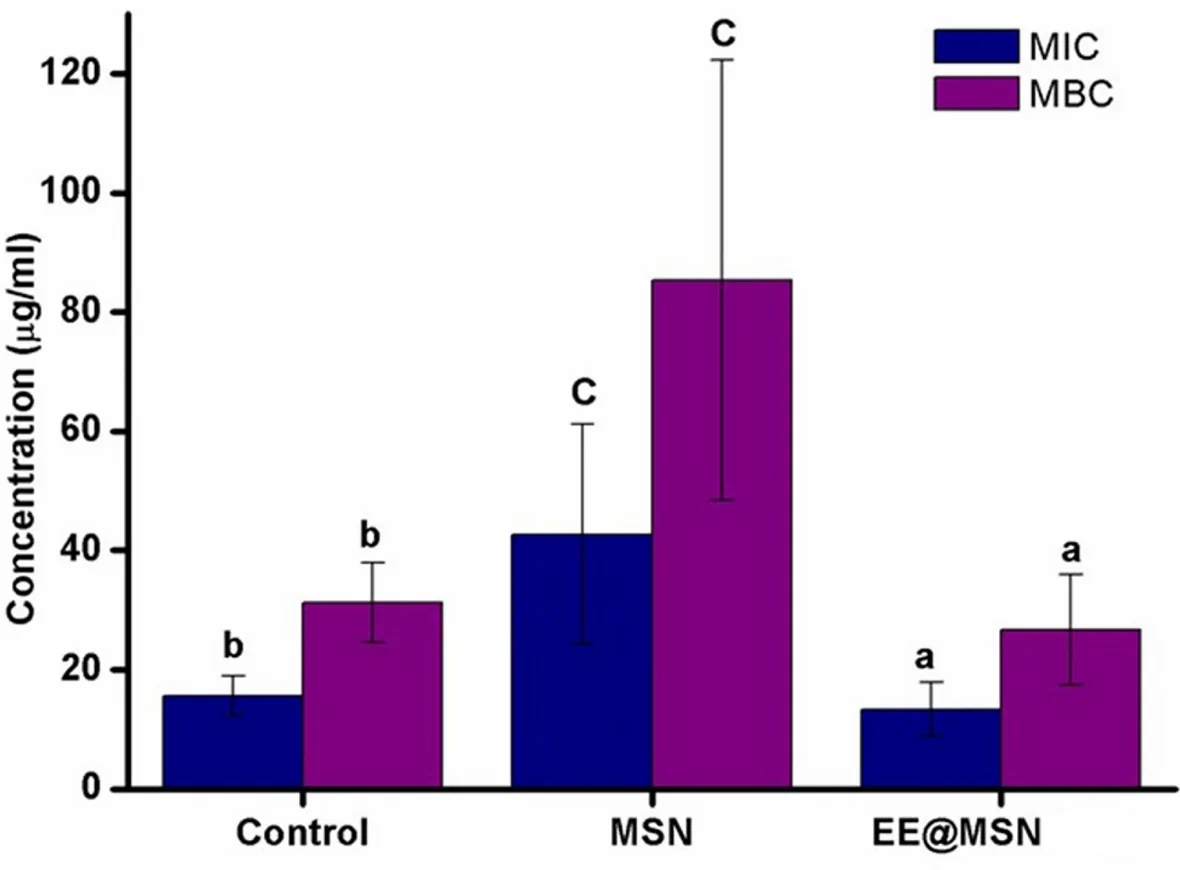
MIC and MBC of MSNs and EE@MSNs against *S. mutans* with chlorhexidine mouthwash control drug. Columns with the same letter show no significant difference at *p* < 0.05, whereas different letters are showing significant difference at *p* < 0.05

The antimicrobial efficacy of the tested materials against *S. mutans* is shown in Fig. 7. The EE@MSNs formulation demonstrated significantly greater antimicrobial activity compared to both the empty MSN carrier and the CHX control. EE@MSNs had the lowest MIC (10 µg/mL) and MBC (20 µg/mL), indicating superior potency. In contrast, MSNs alone showed limited activity (MIC 40 µg/mL; MBC 100 µg/mL).

### Agar diffusion method

**Fig. 8:**
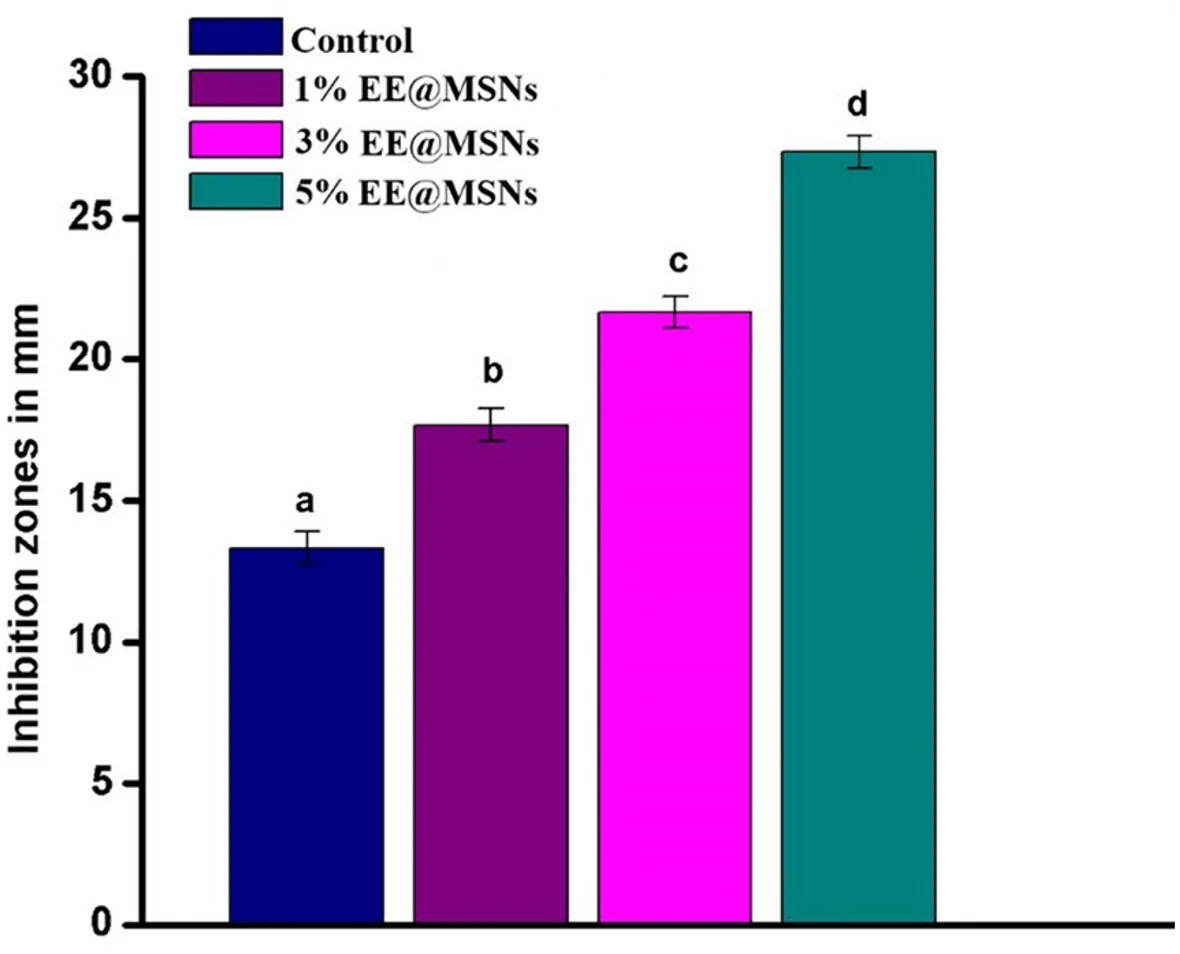
Inhibition zones in (mm) of control and experimental groups. Columns bearing the same letter indicate no significant difference at *p* < 0.05, while different letters indicate significant difference at *p* < 0.05

The measurements of inhibition zones for different groups are illustrated in Fig. 8. The inhibition zones created by the cement disks containing EE@MSNs were significantly larger than the control. A clear dose-dependent relationship was observed, with the 5% EE@MSNs group producing the largest inhibition zone (27 mm), followed by the 3% (22 mm) and 1% (18 mm) groups, all of which were significantly more effective than the control group (15 mm).

### Fluoride release

**Fig. 9:**
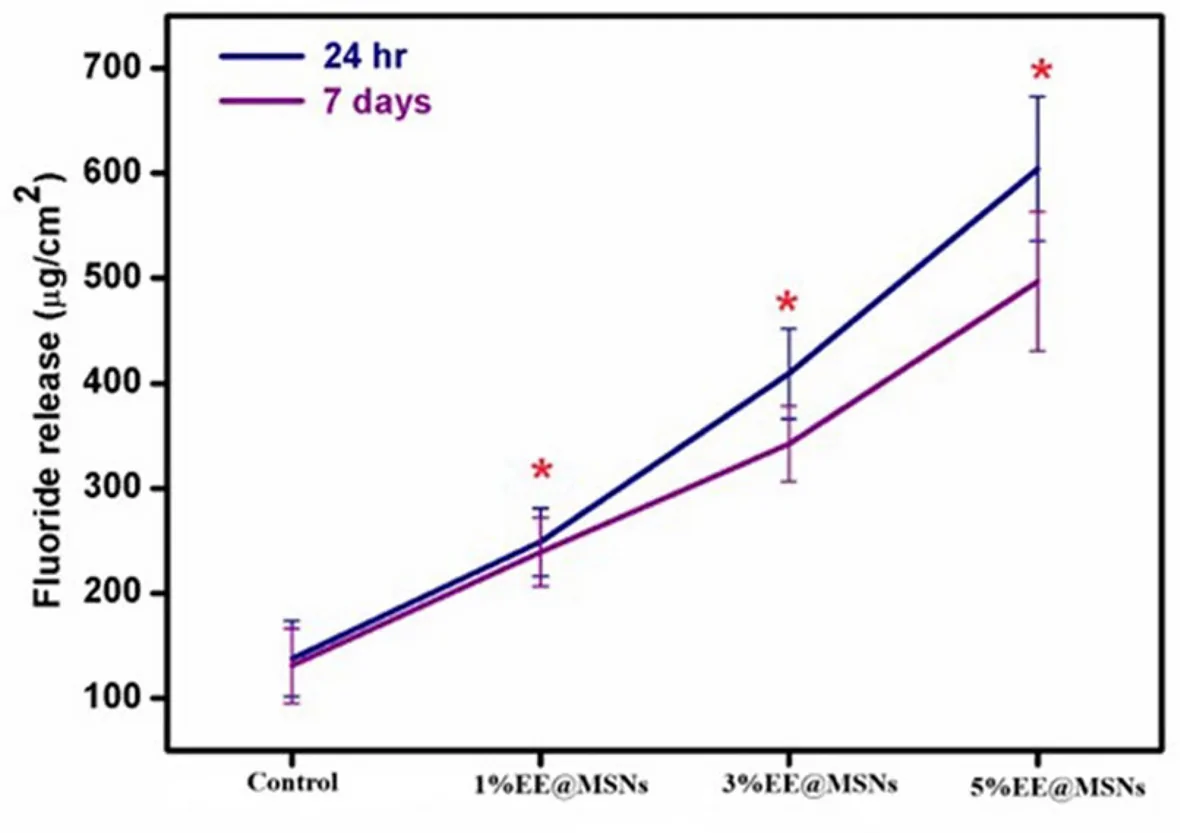
Fluoride release of different tested groups after 24 h and 7 days. *Significant (*p* < 0.05)

Figure 9 demonstrates fluoride release of different groups after 24 h and 7 days. HVGIC modified by different ratios (1, 3, 5% EE@MSNs) recorded significantly higher fluoride release than control groups. Fluoride release of 5% EE@MSNs was significantly higher than 3%EE@MSNs which was also significantly higher than 1%EE@MSNs. The higher percentages of EE@MSNs, the greater fluoride release after different tested periods.

## Discussion

High-viscosity glass-ionomer cement (HVGIC) such as Fuji IX was employed as the preferred material in the ART method. As a result, altering GICs with various antibacterial agents without affecting the other physic-mechanical qualities is recommended [3]. In this investigation, MSNs were prepared and functionalized, and then, eucalyptus extract EE were loaded. Morphological and chemical characterization proved successful functionalization and loading. Both MSNs and EE@MSNs showed high biocompatibility with dental pulp stem cells, maintaining cell viability above 80% at all tested concentrations. According to ISO 10993-5, materials with cell viability ≥ 70% are considered non-cytotoxic, supporting the safety of our formulations for biomedical applications. The encapsulation of eucalyptus extract did not increase toxicity, indicating that the loading process preserves nanoparticle safety and suitability for further development. The results clearly demonstrated that loading eucalyptus extract onto mesoporous silica nanoparticles created a highly effective antimicrobial agent. The superior performance of EE@MSNs, which exhibited lower MIC and MBC values than the gold-standard chlorhexidine, is a key finding of this study. This enhanced activity could be attributed to the synergistic effect of the nanoparticle carrier and the encapsulated herbal extract. The amine-functionalized surface of the MSNs likely improved interaction with the negatively charged bacterial cell wall, while the nanoparticle structure facilitated the controlled release and bioavailability of the active compounds in the eucalyptus extract. This combination allowed for a more potent bactericidal effect than the extract or the nanoparticles alone.

Compressive strength of GIC is important indicator for the functional assessment and clinical evaluation [41]. In this study, significant improvement in compressive strength of 1% and 3% EE@MSNs groups was achieved and this could be explained by that MSNs, act as space fillers, filling up spaces between glass particles. Furthermore, the large surface area of these nanoparticles enabled them to be mechanically interlocked within the matrix resulting into in stress transmission from the matrix itself to the MSNs. These results were in accordance with earlier study on the effectiveness of MSNs as fillers [30]. However, higher ratio (5% EE@MSNs) led to decline in the compressive strength; this might be due to voids created by the nanoparticles’ aggregations which represent weak spots in the structure of the cement. In comparison to other herbal extract modification of GIC, a prior study examined the effect of addition of ethanolic extract of propolis (EEP) to GIC in three ratios (25%, 30%, and 35%) and concluded that its addition in different ratios led to decrease in compressive strength in a proportion-dependent manner [42]. Another study found that miswak extract addition to GIC significantly decreased its compressive strength [43].

Microhardness is an essential quality for restorative materials, as it is associated with a material’s resistance to indentation [44]. Microhardness testing can be used to evaluate the deterioration and durability of restorative materials, to indicate their resistance to wear [45]. This study found a significant enhancement in Vickers microhardness of HVGIC containing 1% and 3 wt. % EE@MSNs, which might provide more evidence for MSNs’ strengthening influence. Microhardness was found to be greater in denser surface structures with lesser voids [30]. A previous study concluded that increasing addition of EEP to GIC significantly improved microhardness of GIC [46].

The present study revealed that HVGIC modified by EE@MSNs successfully inhibited *S. mutans* growth and their inhibitory effect was proportional to the percentages of EE@MSNs addition. The enhanced antimicrobial impact of EE@MSNs groups was consistent with previous study [28]. Other herbal extracts like miswak extract and EGCG Epigallocatechin-3-gallate (EGCG), the polyphenol in green tea were added to GIC and recorded significant improvement in antibacterial inhibition compared to unmodified GIC, but their inhibitory effect was significantly lower than CHX [43, 47].

Recurrent caries is one of the leading causes of restorative failure. Efforts to reduce recurrent caries formation are ongoing in the field of synthetic dental materials. Fluoride release reduces caries activity by acting as a biocide and reducing the solubility of enamel and dentin through fluorapatite generation. Furthermore, fluoride ions interfere with pellicle and plaque production and prevent bacteria growth [16, 37].

In this investigation, specimens were maintained in deionized water, which offers a baseline for fluoride release potential in unstimulated conditions. Deionized water contains no minerals or organic molecules which could interfere with the results [48]. In this investigation, the results showed high amount of fluoride release after 24 h, while a decline in the fluoride release was recorded after 7 days. This is called “Initial Burst Effect” which is desired, because it reduces the survival of germs that remain after caries excavation and induces remineralization of tooth structure [49]. In this study, EE@MSNs addition to glass ionomer has improved its fluoride release on different period measurement and it was proportional to the ratio of EE@MSNs. The enhanced fluoride release was attributed to the content of fluoro-alumino-silicate glass of glass-ionomer cement powder, which was reduced by EE@MSNs introduction, so aqueous polyacrylic acid could more readily react with the glass particles. This led to rapid and aggressive acidic disintegration of the powder particle and subsequently more fluoride release [50]. Also, Zafar et al. [51] reported that the addition of nano-sized particles could enhance fluoride release.

The null hypothesis was rejected as addition of EE@MSNs to HVGIC has improved its mechanical properties at lower ratios, while higher ratios of modification resulted in decline of these properties. While antibacterial and fluoride release were significantly improved by different ratios of modifications.

## Limitations and future studies

The experiments were conducted under static laboratory conditions and did not include an assessment of the effect of thermomechanical aging, which would be necessary to simulate the stresses of the oral environment over time and evaluate the long-term integrity of the modified cement. Furthermore, while the initial fluoride release was measured, the evaluation should be extended to longer periods to understand its sustained therapeutic potential. Crucially, the fluoride recharge and re-release capabilities of the modified GIC, a key property for long-term caries prevention, were not assessed in this study. Finally, this investigation was limited to specific filler percentages; future studies should evaluate a broader range of filler modifications to optimize the material’s antimicrobial and mechanical properties. Addressing these factors is crucial for determining the clinical longevity and full therapeutic potential of the material.

## Conclusion

Although high ratio (5% EE@MSNs) had more effective antimicrobial activity, their negative impact on mechanical properties suggests that lower concentrations (1% and 3%) are more reliable strengthening and antibacterial modification of HVGIC which consequently could improve restoration longevity and achieve potential improvement in ART outcomes.

## Data Availability

All data are available from the corresponding author upon reasonable request.
